# 

**Published:** 1894-07

**Authors:** 


### TABLE OF CONTENTS.

ORIGINAL COMMUNICATIONS. PAGE

Elevation of Teeth in the Sockets. By J. N. FARRAR, M.D., D.D.S., New York City . . . 417

The History and Clinical Appearances of Syphilis, with Especial Reference to the Dangers arising from its Oral Manifestations. By CHARLES M. WHITNEY, M.D., Boston . . . 421

Ocular Headaches. By CHARLES F. STACEY, M.D., Boston . . . 431

A Simple Test for Cocaine in Local Anæsthetics. By MR. H. CARLTON SMITH, Boston . . . 437

Latch-Lock, Lever-Clasp, Plates, and Bridges. By WM. S. DAVENPORT, D D.S., Paris, France . . . 441

Cohesiveness of Tin Fillings. By DR. CHARLES E. FRANCIS, New York 443

REPORTS OF SOCIETY MEETINGS.

New York Odontological Society . . . 444

American Academy of Dental Science . . . 455

Central Dental Association of Northern New Jersey . . . 467

EDITORIAL.

Will We be Known by Another Name? . . . 475

The Annual Conventions . . . 477

The Semi-Centennial of the Discovery of Anæsthesia . . . 478

BIBLIOGRAPHY . . . 478

CURRENT NEWS.

American Dental Association . . . 479

American Dental Society of Europe . . . 479

Special Notice.—Meeting of the American Dental Association at Old Point Comfort, August 7 . . . 480

Illinois State Dental Society . . . 480

